# Sodium Acetate Inhibit TGF-β1-Induced Activation of Hepatic Stellate Cells by Restoring AMPK or c-Jun Signaling

**DOI:** 10.3389/fnut.2021.729583

**Published:** 2021-09-30

**Authors:** Weiwei Li, Mingjuan Deng, Jiahui Gong, Xiaoying Zhang, Shaoyang Ge, Liang Zhao

**Affiliations:** ^1^Key Laboratory of Functional Dairy, College of Food Science and Nutritional Engineering, China Agricultural University, Beijing, China; ^2^Department of Nutrition and Health, Beijing Advanced Innovation Center for Food Nutrition and Human Health, China Agricultural University, Beijing, China; ^3^Inner Mongolia Dairy Technology Research Institute Co., Ltd., Hohhot, China; ^4^Hebei Engineering Research Center of Animal Product, Sanhe, China

**Keywords:** short chain fatty acid, fibrosis, hepatic stellate cells, AMPK, PPARγ

## Abstract

Short-chain fatty acids (SCFAs) are crucial gut microbial metabolites that play a major role in the occurrence and development of hepatic fibrosis (HF). However, the effect of SCFAs on hepatic stellate cells (HSCs), the major pro-fibrogenic cells, is yet undefined. In this study, the effects of three major SCFAs (acetate, propionate, and butyrate) were assessed on the activation of HSCs. LX2 cells were activated with TGF-β1 and treated with sodium acetate (NaA), sodium propionate (NaP), or sodium butyrate (NaB). SCFA treatment significantly reduced the protein levels of α-SMA and the phosphorylation of Smad2 and decreased the mRNA expression of *Acta2/Col1a1/Fn* in cells compared to the TGF-β1 treatment. Among the three SCFAs, NaA revealed the best efficacy at alleviating TGF-β1-induced LX2 cell activation. Additionally, acetate accumulated in the cells, and G protein-coupled receptor (GPR) 43 silencing did not have any impact on the inhibition of LX2 cell activation by NaA. These findings indicated that NaA enters into the cells to inhibit LX2 cell activation independent of GPR43. The results of phosphokinase array kit and Western blot indicated that NaA increased the AMP-activated protein kinase (AMPK) activation and reduced the phosphorylation of c-Jun in cultured LX2 cells, and siRNA-peroxisome proliferator-activated receptor (PPAR) -γ abolished the inhibitory effects of NaA against TGF-β1-induced LX2 cell activation. In conclusion, this study showed that NaA inhibited LX2 cell activation by activating the AMPK/PPARγ and blocking the c-Jun signaling pathways. Thus, SCFAs might represent a novel and viable approach for alleviating HF.

## Introduction

Hepatic fibrosis (HF) is a wound-healing response of the liver to the continuous action of various injury factors, characterized by the net accumulation of extracellular matrix (ECM) or scar (1). A variety of chronic stimuli, including chronic viral infection (hepatitis B or C virus), toxic damage, alcohol abuse (long-standing excessive alcohol consumption), non-alcoholic fatty liver disease (NAFLD)/non-alcoholic steatohepatitis (NASH), autoimmune liver diseases, and metabolic and genetic diseases, could cause HF (2). Subsequently, HF may progress to cirrhosis associated with several complications, such as functional liver failure, hepatic encephalopathy, renal and cardiac disturbances, and hepatocellular carcinoma (HCC) (3). During HF, the ongoing liver injury results in excessive ECM deposition, and abundant Col1a1 and 3 lead to scarring and fibrosis (4). HSCs are the primary source of activated myofibroblasts and portal fibroblasts that drive the fibrogenic process. In response to liver injury, the quiescent, vitamin-A storing HSCs transdifferentiate to contractile, proliferative, and fibrogenic myofibroblasts (5). Then, the cells produce collagen as well as other types of ECM tissue inhibitors of metalloproteinases and matrix metalloproteinases (MMPs) that elicit liver architecture remodeling, thus propagating fibrosis (6). This process is known as HSC activation, and activated HSCs are responsible for about 80% of total fibrillar Col1a1 in the fibrotic liver (7). Therefore, inhibiting HSCs activation is emerging as a promising target for fibrosis alleviation.

Currently, the literature is focused on the correlation between gut and liver. The liver receives most of its blood flow (70%) from intestinal vascularization, and hence, is constantly exposed to nutrients, toxins, and products of the gut microbiota (8). Moreover, the gastrointestinal tract receives a liver product in the form of bile acid (9). This functional bidirectional correlation between the liver and gastrointestinal tract is known as the gut-liver axis (GLA) (8). The dysregulation of the microbiota has been confirmed in patients with HF (10). Several studies reported that the ratio of *Firmicutes/Bacteroidetes* in microbiota was correlated with lipid accumulation and HF in both human and animal models (11, 12). In a group of biopsy-proven NAFLD patients, a higher abundance of *Bacteroides* were observed in fibrosis subjects compared to those without fibrosis, suggesting a correlation between *Bacteroides* and HF severity (13). Previous studies also showed that patients with cirrhosis had a decrease in commensal SCFA-producing bacteria, especially butyrate-producing bacteria, including Lachnospiraceae and Ruminococcaceae (14). Accumulating evidence indicated a balance in the microbial community by supplementing probiotics or prebiotics, which alleviated HF. Gadallah et al. (15) demonstrated that treatment with the probiotic mixture and prebiotic insulin suppressed the expression of inflammatory IL-6 and reduced the level of fibrotic TGF-β1 and α-SMA markers in the experimental rats. In humanized mice, dietary fiber altered the intestinal microbiota composition produced abundant SCFAs and exerted a protective effect on mouse HF (16). These studies suggested that gut microbiota had a tight correlation with HF, and SCFAs could be major regulators of these processes.

SCFAs are the main microbial metabolites of dietary fibers of gut microbiota. Acetate, propionate, and butyrate are the most abundant SCFAs in the human gut (17). Importantly SCFAs are not only metabolic substrates but also signaling molecules that regulate liver metabolism (18). A previous study indicated that fructo-oligosaccharide treatment increased intestinal SCFAs and improved hepatic steatosis, inflammatory cell infiltration, and hepatocyte ballooning of NASH mice (19). Takayama et al. (20) showed that feeding partially hydrolyzed guar gum increases butyrate, acetate, and propionate in the gut and attenuates liver inflammatory markers (TNF-α and MCP-1) and fibrogenic markers (Col1a1 and α-SMA) in mice. These results indicated that regulating the level of gut SCFAs alleviates NASH-related fibrosis via complicated processes. In addition, direct oral supplementation of butyrate remarkably alleviates the liver fibrosis stage by decreasing the expression of inflammatory maker protein (MyD88) and alleviating liver steatosis and lipid degeneration of NASH mice, interestingly, the inhibition of iNOS may be involved in the potential mechanism for butyrate alleviating NASH (21). However, only a few studies have focused on the protective effects of specific SCFAs on the development of HF, especially in HSC activation; nonetheless, the related mechanisms are not yet elucidated.

In this study, TGF-β1-activated HSCs were employed to evaluate the effects of three dominant SCFAs, NaA, NaP, and NaB, on fibrotic markers. The mechanisms related to the NaA-induced inhibition of HSC activation were assessed using protein antibody chip and RNA interference (RNAi) in HSCs.

## Materials and Methods

### Cell Culture

HSC-LX2 cells were purchased from Stem Cell Bank, Chinese Academy of Sciences (Shanghai, China) and cultured in Dulbecco's modified Eagle's medium (DMEM; Gibco, Grand Island, NY, USA) containing 2% fetal bovine serum (FBS, Gibco) and 100 U/mL penicillin and streptomycin (Beyotime, Beijing, China) in a 5% CO_2_ incubator at 37°C (22). To study the effects of SCFAs on TGF-β1-induced fibrogenesis, LX2 cells were seeded in six-well plates (Corning, Corning, NY, USA) in 2 mL of DMEM medium at a density of 1 × 10^6^ cells/mL. After achieving 70–80% confluency, the cells were subjected to 12 h of serum starvation, while the control group was incubated with fresh DMEM and the cells in the other groups were incubated with DMEM containing 2.5 ng/mL TGF-β1 (PeproTech, Rocky Hill, NJ, USA) and varying concentrations of NaA (0.1 or 1 mM) for an additional 48 h. Next, we selected the concentration based on previous toxicological studies in LX2 cells, especially those related to fibrogenesis. Subsequently, the cells were used for Western blot analysis and/or harvested for total RNA isolation.

### Cell Cytotoxicity Assays

An Enhanced Cell Counting Kit 8 Assay (Beyotime, Shanghai, China) was used to determine cell cytotoxicity of SCFAs (23). LX2 cells were seeded at a density of 5 × 10^3^ per well onto flat-bottom 96-well culture plates (Corning). Cells were treated by NaA, NaP or NaB (0 to 1 mM) as mentioned earlier. The absorbance values of viable cells were finally determined at 450 nm using a microplate spectrophotometer (BioTek, Winooski, VT, USA). The cell inhibitory rates were calculated using the following formula: Cell inhibition rate (%) = (1 – A_450_ (sample) / A_450_ (control)) × 100.

### Western Blot Analysis

Total protein was isolated from cultured cells using lysis buffer supplemented with protease and phosphatase inhibitors. The protein concentration was measured using a protein assay kit (Bio-Rad, Hercules, CA, USA). An equivalent of 30 μg (24) protein extract was separated by sodium dodecyl sulfate-polyacrylamide gel electrohoresis (SDS-PAGE) and transferred to polyvinylidene difluoride membranes (PVDF). The membranes were incubated with primary antibodies: phosphorylated AMPK alpha (p-AMPKα), AMPKα, glyceraldehyde-3-phosphate dehydrogenase (GAPDH), phosphorylated c-Jun (Ser63) (p-c-Jun), c-Jun, phosphorylated Smad2 (p-Smad2), Smad2, β-Actin (CST, Danvers, MA, USA), PPARα, and PPARγ (Abcam). After incubation with a goat anti-rabbit horseradish peroxidase-conjugated secondary antibody (Beyotime, Shanghai, China) at a dilution of 1:10,000 for 1 h, the proteins were visualized using a Luminata Forte Enhanced Chemiluminescence Kit (Millipore, Billerica, MA, USA). The band intensities were analyzed using Quantity One analysis software.

### Quantitative Real-Time Polymerase Chain Reaction

Total RNA was extracted using TRIzol Reagents (Ambion, Austin, TX, USA) and subjected to reverse transcription using a Prime Script RT-PCR kit (TaKaRa). qRT-PCR was carried out using SYBR Premix Ex Taq (TaKaRa) on the Light-Cycler 480 (Roche Diagnostics GmbH) and analyzed by the software (25). The primers were synthesized by Sangon Biotech, China (Supplementary Table 1). The fluorescence data of the target genes were analyzed by the 2^−ΔΔCt^ method for relative quantification using *Actin* or *GAPDH* as an internal control.

### Measurement of Intracellular Sodium Acetate Concentration

SCFA extraction: 10^8^ cells were mixed with 2 mL extraction reagent (water:phosphoric acid = 1:3) and homogenized for 20 s at 6,500 × *g* using a Precellys 24 homogenizer (Bertin Technologies, Montigmyle Bretonnexux, France). The cell extract was prepared by adding 2 mL ether on ice for 10 min, followed by centrifugation at 4,000 × *g* for 20 min (26). The remaining aqueous layer was further extracted with ether. Subsequently, the ether layers were pooled and diluted to 2 mL. Then, samples were subjected to gas chromatography-mass spectrometer (GC-MS) analysis using a 7890B gas chromatograph/5977 mass selective detector (Agilent Technologies, Santa Clara, CA, USA) with an HB-5 ms capillary column (30 m × 0.25 mm × 0.25 μm film thickness) (Agilent Technologies), with helium as carrier gas at a constant flow rate of 1 mL/min. Samples (0.5 μL) were injected using a pressure pulsed split mode (10 psi) with a split ration of 10:1. The initial column oven temperature was 100°C for 1 min, and then increased to 120°C at a rate of 10°C/min and held for 5 min, then increased to a final temperature of 220°C at a rate of 30°C/min and held for 3 min. The total run time was 14.3 min and all the data was collected in full scan mode with a mass range of 40–300 *m/z* (27). Pure water was used as a blank sample to correct the background. A blank sample was processed similar to that of cells samples. The corrected peak area of acetic acid was calculated by the peak areas of samples minus that of the blank sample detected under the same conditions.

### Small Interfering RNA Transfection

siRNA targeting GPR43, PPARγ, or control siRNA was synthesized by Biolino Biotech (Tianjin, China) (Supplementary Tables 2, 3). Transfections were performed using the Lipofectamine^®^ 2000 RNAiMax reagent (Invitrogen, Karlsruhe, Germany) following the manufacturer's instructions. As described previously, cells were treated with three concentrations of SCFAs and TGF-β1 for 48 h after 24 h post-transfection (28). The downregulation of the GPR43 or PPARγ targeted by siRNA was confirmed by RT-PCR.

### Phosphokinase Array

Relative protein phosphorylation levels were obtained by profiling 43 specific phosphorylation sites (Supplementary Table 4) using the Proteome Profiler Human Phosphokinase Array Kit (ARY003B, R&D Systems), according to the manufacturer's instructions. Briefly, the cells were rinsed with phosphate-buffered saline (PBS), resuspended in lysis buffer (1 × 10^7^ cells/mL), and agitated at 4°C for 30 min (29). The array membranes were blocked with 1.0 ml of Array Buffer one and incubated with 500 μg cell lysates at room temperature for 1 h. Next, cocktails of biotinylated detection antibodies were added and samples were incubated at room temperature for 2 h. Phosphorylated proteins were revealed using streptavidin-HRP/chemiluminescence substrate (sc-201696, Santa Cruz Biotechnology, Santa Cruz, CA, USA) and autoradiography films. The resulting spots were scanned, and images were quantified using the Image Lab software (Bio-Rad).

### Measurement of Intracellular AMP:ATP Ratio

Following exposure to acetate, LX2 cells were removed the supernatant and washed three times with ice-cold PBS, and then added 3 mL of ice-cold aqueous acetonitrile (50%, v/v) (VWR) to break cells to extracte intracellular nucleotides. The resulting extract was maintained on ice for 10 min, followed by centrifugation at 14,000 × g for 1 min at 0°C. The extract of cell contents was collected and dried using a refrigerated SpeedVac (Savant). The dried extract was resuspended in 240 μL of deionized water and filtered using a 0.22-μm syringe filter unit for high-performance liquid chromatography (HPLC) analysis (30). The chromatographic separation and analysis were performed on an Agilent system (1,200 series), equipped with a diode-array detector and a C18 reverse-phase column (Kromasil, 5 μm, 100 Å; 4.6 × 150 mm) at a flow rate of 1 mL/min and a linear gradient of acetonitrile (0–7%) in 10 mM triethylammonium acetate buffer (Glen Research) over 20 min. AMP and ATP were identified based on their retention times.

### Statistical Analyses

Numeric data were presented as mean ± SD. Statistical significance was determined using the one-way ANOVA followed by LSD post-tests with SPSS 22.0. Differences were considered significant at a two-tailed *p*-value < 0.05.

## Results

### NaA-H Ameliorates TGF-β1-induced LX2 Cell Activation

**Figure 1:**
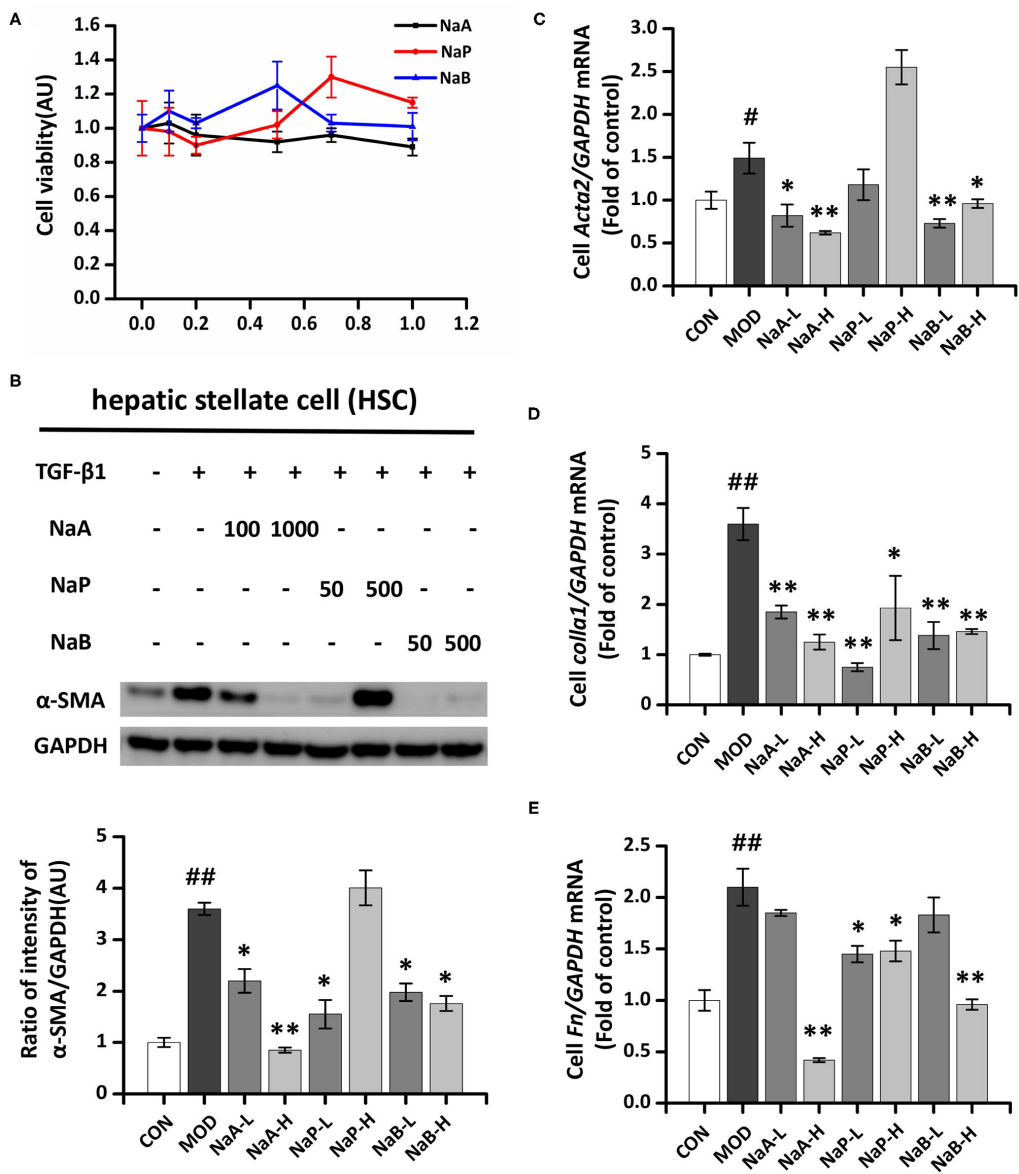
Effects of SCFAs on TGF-β1 induced LX2 cells activation. **(A)** SCFAs cytotoxic effects. After serum starvation for 12 h, a CCK8 assay was performed for LX2 cells treated with SCFAs at doses of 0–1 mM for 48 h. **(B)** The protein expression of α-SMA was assessed by the Western blot. LX2 cells were treated with or without 2.5 ng/ml TGF-β1 and 1 mM of SCFAs for 48 h. **(C–E)** Real-time PCR was used to evaluate the mRNA expressions of *Acta2, Col1a1*, and *Fn*. LX2 cells were treated with or without 2.5 ng/ml TGF-β1 and 1 mM of SCFAs for 48 h. LX2 cells were treated as detailed in the section Materials and Methods. For all bar graphs, data are the mean ± SD, ^#^*p* < 0.05, ^##^*p* < 0.01, as compared with CON, and ^*^p < 0.05, ^**^p < 0.01, as compared with MOD. The significant difference was assessed using the one-way ANOVA followed by LSD post-tests. Control group (CON), group model cell treated with a TGF-β1 (MOD), low dose of sodium acetate (NaA-L), high dose of sodium acetate (NaA-H), low dose of sodium propionate (NaP-L), high dose of sodium propionate (NaP-H), low dose of sodium butyrate (NaB-L), high dose of sodium butyrate (NaB-H), arbitrary unit (AU).

A CCK-8 assay was performed to evaluate the potential cytotoxic effect of SCFA on HSCs. The results showed that after treatment with the maximum concentration of SCFAs (1 mM) for 48 h, >80% of the LX2 cells survived (Figure 1A), indicating that SCFAs did not affect cell viability. Regarding the antifibrotic effects of the three different SCFAs on TGF-β1-induced HSCs activation, the protein expression level of α-SMA, the marker of fibrogenesis, was analyzed by Western blot. Compared to the control group, TGF-β1 treatment significantly elevated α-SMA in the MOD group, which indicated LX2 cell activation by TGF-β1. SCFA treatment significantly inhibited the overexpression of α-SMA in activated LX2 cells except high-dose of NaP compared to the MOD group. Specifically, high-dose of NaA (NaA-H) or NaB (NaB-H) reduced 72.2 or 53.7% α-SMA levels in cells compared to the MCD group (Figures 1B,D). NaA-H generated maximum efficacy at reducing α-SMA among the SCFA treatment groups. Similar results were also observed in gene expression levels of *Acta2*, the α-SMA-coding gene. TGF-β1 treatment increased the levels of *Acta2* compared to the control group, while treatment with high-dose of SCFAs inhibited *Acta2* levels, which were 57.7% (NaA-H), 26.4% (NaB-H), and 19.8% (NaP-H) lower than those in the MOD group, respectively (Figure 1C). These results confirmed that NaA-H had the best inhibitory effect on LX2 cell activation. Moreover, SCFAs also suppressed the overexpression of fibrosis marker genes *Col1a1* and *Fn* in TGF-β1-activated LX2 cells. For *Col1a1* gene expression, a high dose of NaA or NaB showed significant inhibition compared to the MOD group (Figure 1D). For *Fn* gene, all high-dose groups of three SCFAs represented significant inhibition to the MOD group (Figure 1E). The results indicated that SCFA treatment ameliorated TGF-β1-induced LX2 cell activation. NaA-H revealed the best efficacy at alleviating TGF-β1-induced LX2 cell activation relative to propionate and butyrate. Therefore, NaA-H was selected for further experiments.

### NaA Treatment Inhibited TGF-β1/Smad2 Signaling in LX2 Cells

**Figure 2:**
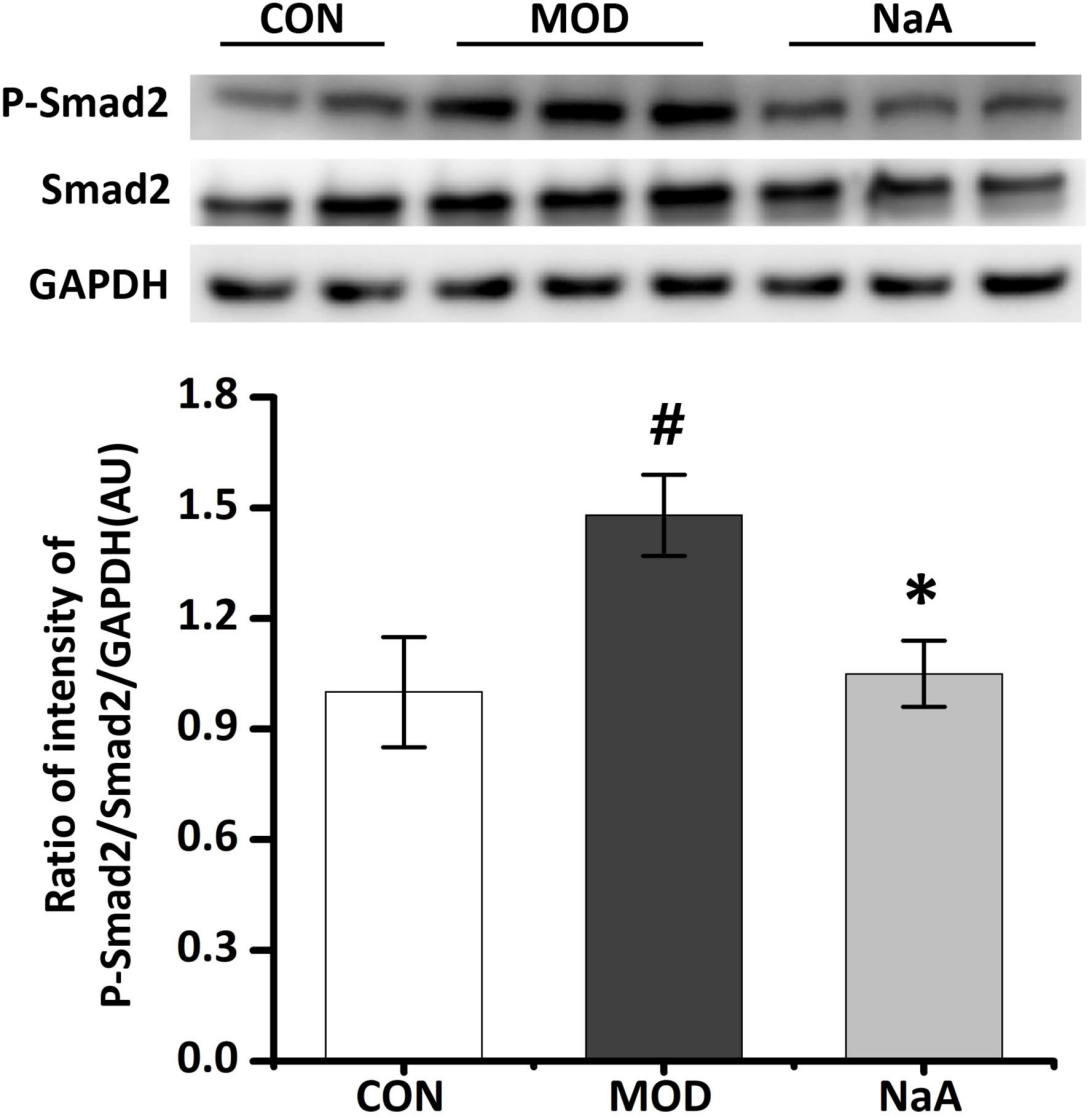
NaA inhibited TGF-β1 induced HSCs activation in LX2 cells. LX2 cells were treated with or without 2.5 ng/ml TGF-β1 and 1 mM of NaA for 48 h. Western blot was used to evaluate the phosphorylation of Smad2. LX2 cells were treated as detailed in the section Materials and Methods. For all bar graphs, data are the mean ± SD, ^#^*p* < 0.05, as compared with CON, and ^*^p < 0.05, as compared with MOD. The significant difference was assessed using the one-way ANOVA followed by LSD post-tests. Control group (CON), group model cell treated with a TGF-β1 (MOD), sodium acetate (NaA), arbitrary unit (AU).

As the downstream cascade, Smad2 signaling plays a key role in TGF-β1-induced HSC activation. Compared to the control group, TGF-β1 markedly enhanced the phosphorylation level of Smad2 protein, while NaA treatment significantly ameliorated the phosphorylation of Smad2 (Figure 2). These phenomena were consistent with previous results, indicating that NaA inhibits the phenotype and key signaling pathways.

### NaA Entered Into Cells to Inhibit LX2 Cell Activation Independent of GPR43

**Figure 3:**
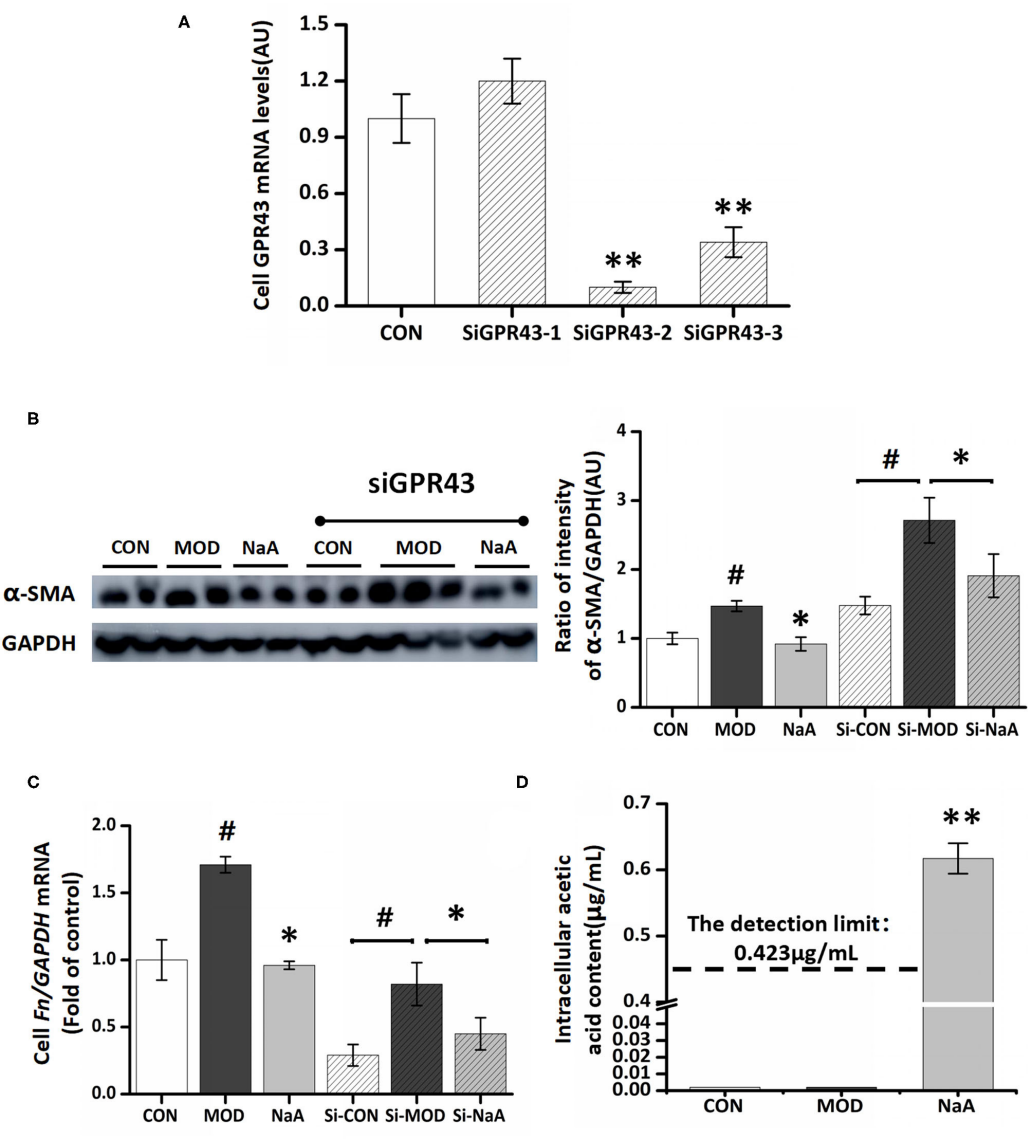
GPR43 possessed limiting ability in the process of NaA inhibited TGF-β1 induced HSCs activation. **(A)** LX2 cells were cultured and transfected with 50 nM Lipofectamine^®^ 2000 RNAiMax reagent and GPR43 siRNA, selected the sequences with the best silence efficiency. **(B)** Western blot was used to evaluate the protein of α-SMA. LX2 cells were treated with or without 2.5 ng/ml TGF-β1 and 0–1 mM of NaA for 48 h at 24 h post-transfection. **(C,D)** Real-time PCR was used to evaluate the mRNA expression of *Col1a1*. LX2 cells were treated with or without 2.5 ng/ml TGF-β1 and 0–1 mM of NaA for 48 h at 24 h post-transfection. LX2 cells were treated as detailed in the section Materials and Methods. For all bar graphs, data are the mean ± SD, ^#^*p* < 0.05, as compared with CON, and ^*^p < 0.05, ^**^p < 0.01, as compared with MOD. The significant difference was assessed using the one-way ANOVA followed by LSD post-tests. Control group (CON), group model cell treated with a TGF-β1 (MOD), sodium acetate (NaA), arbitrary unit (AU).

Next, we examined whether GPR43, a promising receptor of acetate, participated in the LX2 cell inhibition by acetate. GPR43 siRNA was used to silence its target mRNA, specifically in LX2 cells. Herein, we designed three siGPR43 sequences; the silencing efficiency of the siGPR43-2 sequence was 90.6%, and hence, it was selected for downstream experiments in LX2 cells (Figure 3A). The results showed that GPR43 silencing did not have an impact on the decreased expression of α-SMA protein and *Col1a1* mRNA by NaA (Figures 3B,C), indicating that NaA inhibited the activation of LX2 cells via pathways independent of GPR43. In addition to G proteins, histone deacetylase (HDAC) enzymes may also act as target sites of SCFA. SCFAs exert their effects by binding to metabolite-sensing GPR41, GPR43, and GPR109A or epigenetically via HDAC modulation. Since the GPR43 receptor does not influence the function of acetate, the impact of acetate on HDAC was further investigated and speculated to be involved in the beneficial effects of SCFAs. Compared to the control group, TGF-β1 or NaA treatment did not significantly alter the expression of HDAC2, HDAC4, HDAC5, HDAC6, HDAC7, HDAC8, HDAC9, and HDAC11. Although NaA treatment had a significant effect on the expression of HDAC1, HDAC3 and HDAC10, TGF-β1 treatment had no influence on the expression of these genes indicating they were not involved in activation of LX2 cells. Therefore, the results showed that HDAC did not participated in NaA inhibiting the activation of LX2 cells (Supplementary Figure 1). These results indicated that GPR43 or HDAC might not participate in the inhibition of NaA-activated HSCs.

On the other hand, NaA may enter into the cells and have some effects. To verify this hypothesis, we used a GC-MS to detect the intracellular acetate content. Compared to the control and model groups, treatment with NaA (1 mM) caused a significant increase in the content of acetate, while the other two groups were lower than the detection limit (0.42 μg/mL) (Figure 3D). The present results showed that inhibiting HSC activation by acetate might be achieved by NaA entering into the cell.

### NaA Attenuated LX2 Cells Activation in an AMPKα- and c-Jun-Dependent Manner

**Figure 4:**
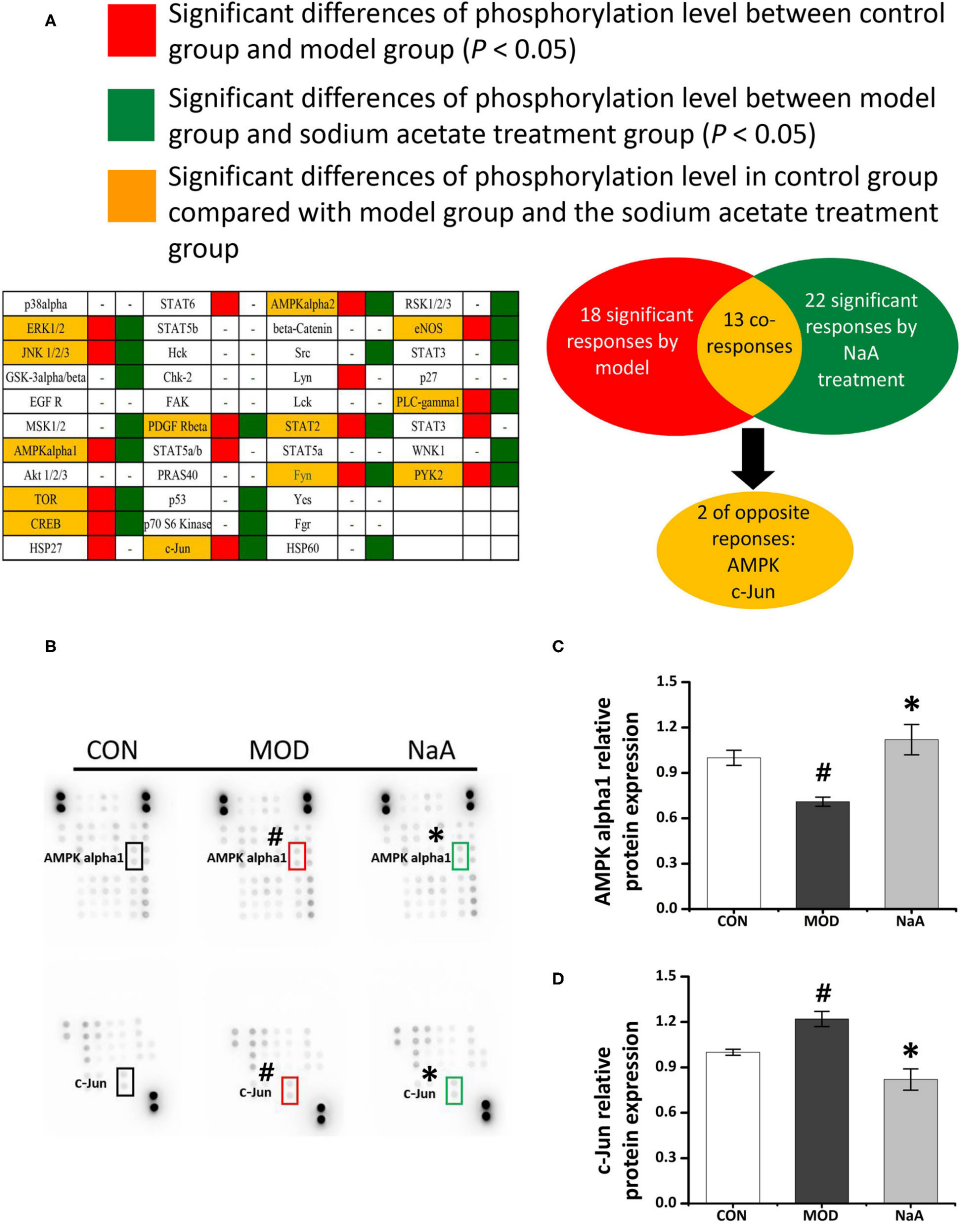
Potential targets of NaA. LX2 cells were treated with or without 2.5 ng/ml TGF-β1 and 0–1 mM of NaA for 48 h. After quantitative analysis of the results using gray analysis software. **(A)** The component-target network. Phosphorylation analysis of 43 potential targets. 43 protein contains a protein called p53, protein p53 has three phosphate sites (s15, s392, and s46), we will uniform them as p53. The red represents to compare with the CON, the model is significantly different, the green represent to the NaA treatment group is significantly different from MOD, the yellow part means that both MOD and NaA treatment group are significant. **(B–D)** Phospho-kinase array screening. Differentially expressed phosphor-proteins are labeled by frames, two types with opposite trends, namely AMPKα and c-Jun amino-terminal kinase. LX2 cells were treated as detailed in the section Materials and Methods. For all bar graphs, data are the mean ± SD, ^#^*p* < 0.05, as compared with CON, and ^*^p < 0.05, as compared with MOD. The significant difference was assessed using the one-way ANOVA followed by LSD post-tests. Control group (CON), group model cell treated with a TGF-β1 (MOD), sodium acetate (NaA), arbitrary unit (AU).

To analyze the potential mechanism of NaA on the activation of LX2 cells induced by TGF-β1, a phosphokinase array kit containing 43 kinase phosphorylation targets was used (Supplementary Table 2). TGF-β1 induced significant changes in the phosphorylation level of 18 proteins compared to the control group. NaA treatment altered the phosphorylation level of 22 proteins compared to the MOD group. Herein, the significant changes in the phosphorylation of the protein effectuated by both TGF-β1 and NaA treatment were investigated (Figure 4A). We found that the phosphorylation of AMPKα was decreased when LX2 cells were exposed to a medium containing TGF-β1, while NaA supplementation enhanced the phosphorylation of AMPKα significantly (Figures 4B,C). On the other hand, phosphorylation of c-Jun was significantly upregulated in TGF-β1-activated HSCs, and NaA treatment restored the increasing trend (Figures 4B,D). These findings indicated that AMPKα and c-Jun pathways were highly related to the inhibition of NaA-activated LX2 cells.

**Figure 5:**
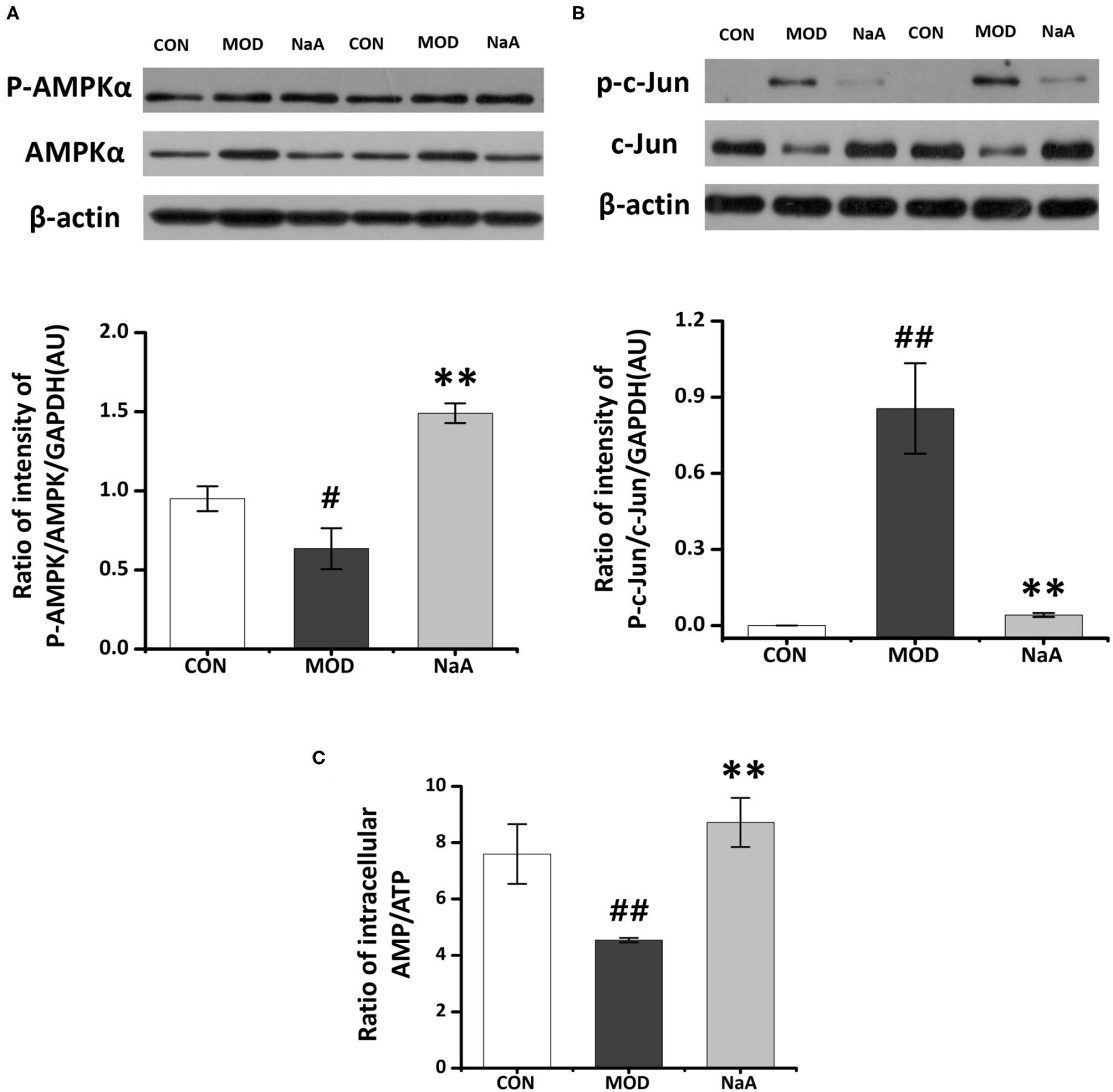
Research on the specific mechanism of NaA on the activaon of LX2 cells. LX2 cells were treated with or without 2.5 ng/ml TGF-β1 and (0–1) mM of NaA for 48 h at 24 h post-transfection. **(A)** HPLC was used to evaluate Intracellular AMP: ATP. **(B,C)** Western blot was used to evaluate phosphorylation of AMPKα and c-Jun. LX2 cells were treated as detailed in the section Materials and Methods. For all bar graphs, data are the mean ± SD, ^#^*p* < 0.05, ^##^*p* < 0.01, as compared with CON, and ^**^p < 0.01, as compared with MOD. The significant difference was assessed using the one-way ANOVA followed by LSD post-tests. Control group (CON), group model cell treated with a TGF-β1 (MOD), sodium acetate (NaA), arbitrary unit (AU).

Next, we employed Western blot to verify the ChIP data and confirmed that the phosphorylation of AMPKα was significantly decreased in LX2 cells after short-term exposure to 2.5 ng/mL TGF-β1, which was increased after NaA addition (Figure 5A). Also, NaA treatment significantly reduced TGF-β1-induced phosphorylation of c-Jun (Figure 5B), which was consistent with the ChIP results. Furthermore, we used HPLC to measure the intracellular AMP/ATP content and found that the AMP/ATP ratio decreased in the MOD group but was restored in the NaA treatment group, which might contribute to the changes in the AMPKα phosphorylation level (Figure 5C). These results indicated that a high dose of NaA was capable of inhibiting TGF-β1-induced cell activation via AMPKα and c-Jun pathways.

### NaA-Alleviated LX2 Cell Activation Is Related to PPARγ Pathway

**Figure 6:**
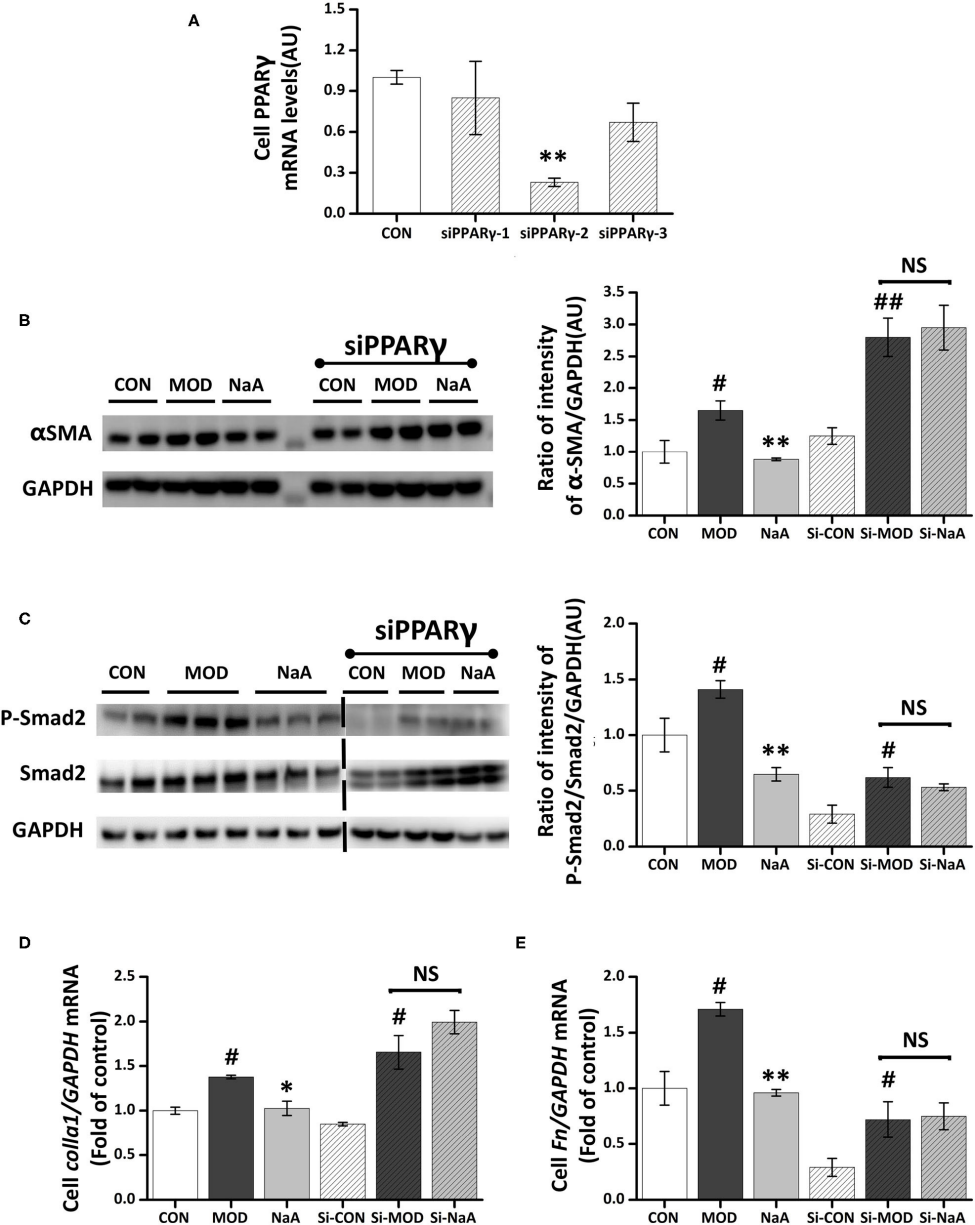
NaA-mediated anti-fibrosis effects were partially reversed by siRNA-PPARγ. LX2 cells were cultured and transfected with 50 nM Lipofectamine^®^ 2000 RNAiMax reagent and PPARγ siRNA. **(A)** Real-time PCR was used to evaluate the mRNA expression of PPARγ, selected the sequences with the best silence efficiency. **(B,C)** Western blot was used to evaluate the protein of α-SMA and phosphorylation of Smad2. LX2 cells were treated with or without 2.5 ng/ml TGF-β1 and 0, 1 mM of NaA for 48 h at 24 h post-transfection. **(D,E)** Real-time PCR was used to evaluate the mRNA expressions of *Col1a1* and *Fn*. LX2 cells were treated with or without 2.5 ng/ml TGF-β1 and 0–1 mM of NaA for 48 h at 24 h post-transfection. LX2 cells were treated as detailed in the section Materials and Methods. For all bar graphs, data are the mean ± SD, ^#^*p* < 0.05, ^##^*p* < 0.01, as compared with CON, and ^*^p < 0.05, ^**^p < 0.01, as compared with MOD. The significant difference was assessed using the one-way ANOVA followed by LSD post-tests. Control group (CON), group model cell treated with a TGF-β1 (MOD), sodium acetate (NaA), arbitrary unit (AU).

We also explored whether PPARγ, the potential target of SCFA for HF, is involved in the protective effect of SCFAs in HSC activation. The roles of PPARγ in the inhibition of acetate-activated LX2 cells were verified using PPARγ silencing. The expression of PPARγ in the silenced cells was decreased (Figure 6A), and siPPARγ-2 represented the best silencing efficiency. Western blot showed that the inhibition of α-SMA (Figure 6B) and phosphorylation of Smad2 (Figure 6C) by SCFAs were significantly disrupted by siRNA-PPARγ. Moreover, siRNA-PPARγ abolished the inhibitory effects of SCFAs against TGF-β1 induced increase in the gene expression of *Col1a1* and *Fn* (Figures 6D,E). Taken together, these data indicated that the inhibition of HF by SCFAs was dependent on PPARγ.

## Discussion

This study evaluated the potential antifibrotic effects of three dominant SCFAs, acetate, propionate, and butyrate, based on TGF-β1-activated LX2 cells. NaA represented the best efficacy at inhibiting fibrotic markers, indicating the ability to alleviate HSCs activation. Furthermore, the underlying mechanisms were elucidated. We found that NaA could enter into cells to inhibit HSC activation rather than through GPR43 receptor or act as an inhibitor of HDAC. Finally, we demonstrated NaA regulates AMPKα/PPARγ and c-Jun signaling pathways, which further block TGF-β1/Smad2 to suppress the activation of LX2 cells. To the best of our knowledge, this is the first study to investigate the direct effects of different SCFAs on activating HSCs and revealing the underlying mechanisms.

As major gut microbial metabolites, SCFAs showed their direct or indirect influence on the gut-liver communications and played a role in liver functions (22). However, inconsistent effects of different SCFAs on liver functions were observed previously. Previous studies demonstrated that SCFAs contributed to obesity and liver steatosis as they provide ~10% of daily caloric consumption and might enhance nutrient absorption by promoting the expression of glucagon-like peptide 2 (GLP-2), obesity, and liver steatosis that could trigger liver inflammation and NAFLD, as well as HF (31). Moreover, another study showed that acetate activated the parasympathetic nervous system resulting in increased ghrelin secretion and glucose-stimulated insulin secretion (GSIS), which in turn generated ectopic lipid deposition and insulin resistance in the liver (32). Conversely, accumulating evidence showed that SCFAs act through GPR41 and GPR43 in L cells to promote peptide YY (PYY) and GLP-1 and alter satiety and energy intake, which further alleviates obesity or NAFLD indirectly (33). SCFAs also regulate hepatic metabolism by altering hepatic AMPK phosphorylation and expression of PPARα target genes involved in free fatty acids (FFAs) oxidation. Thus, SCFAs might act both directly and indirectly to alleviate hepatic metabolism disorder via complicated processes (17). HF was closely associated with various types of chronic inflammatory damage in the liver as a compensatory pathophysiological process, especially for steatosis or steatohepatitis. The activation of HSCs had been confirmed to play a critical role in HF (6). Currently, there is no evidence to show how exogenous SCFAs directly affect HF. To address whether SCFAs improved HF symptoms and which SCFAs were the most effective, we provided primary data concerning the direct effects of acetate, propionate, and/or butyrate on alleviating TGF-β1-induced LX2 cell activation. Among these, high-dose sodium acetate (1 mM) was considered efficacious in alleviating LX2 cell activation. Similarly, in our previous study, NaA displayed maximum efficacy at decreasing liver steatosis (18), i.e., supplementation of SCFA is beneficial for managing HSC activation. We also observed that high dose of propionate increased level of α-SMA and related gene (*Acta2*), but have no influence on *Colla1* and *Fn*. These results suggested high dose of propionate might induce the fibrosis. However, few studies provided similar evidence, except Lee et al. (34) showed propionate level in stool samples increased with worsening fibrosis severity (level of fibrogenic genes and proteins, such as α-SMA) of non-obese NAFLD patients. We noticed that high dose propionate (500–1,000 μmol/L) stimulated viability of LX2 cells (Figure 1A). Since we speculated that induced expression of α-SMA by high dose propionate may related high cell viability by the treatment of propionate. The clear mechanism need to be elucidated in furture studies.

The mechanisms of acetate inhibiting HSCs activation were further investigated in LX2 cells. The biological responses of acetate on host cells result from the inhibition of HDAC or the activation of GPRs, such as GPR41 and GPR43 (32). GPR43 has been identified in the colon, as well as in adipocytes, hepatocytes, enteroendocrine cells, and immune cells (polymorphonuclear cells and macrophages) (35). Next, we assessed whether acetate activates GPR43, the membrane FA receptor for which acetate had the highest affinity, during the HSC suppression (36). In contrast to expectations, the decrease in GPR43 by RNA silencing could not attenuate acetate-altered expression of α-SMA protein and Col1a1 mRNA. Then, we investigated whether acetate altered HSC activation by inhibiting HDAC in LX2 cells, while acetate and/or TGF-β1 treatment did not have an impact on the expression of HDAC mRNA, indicating that NaA inhibited the activation of HSCs via pathways independent of GPR43 or HDAC. On the other hand, the concentration of intracellular acetate was significantly increased in LX2 cells treated by NaA, indicating that acetate entered the cells and affected the intracellular signals. This phenomenon was consistent with the previous report, wherein SCFAs attenuate intestinal inflammation by entering Caco-2 cells (37) through monocarboxylate transporter 1 (MCT1) or sodium monocarboxylate transporter 1 (SMCT1) transport protein and act independently of GPR43 or HDAC (26). Similarly, another study indicated that acetate was absorbed mainly by passive diffusion, accumulated inside m-ICcl2 cells, and stimulated lipid consumption in enterocytes (30). The increasing intracellular concentration of NaA demonstrated that NaA partially inhibits LX2 cell activation by entering the cells.

Acetate might directly interact with intracellular signaling molecules to regulate HSCs activation. However, the molecular mechanisms underlying this effect are yet to be elucidated. Herein, we used antibody-based array kits to analyze the mechanism by which acetate inhibited LX2 cell activation. We found that acetate might inhibit HSC activation through AMPKα and c-Jun pathways. AMPK acted as the primary sensor of cellular energy status and was closely related to HF (38). A previous study showed that the loss of AMPK exaggerates the pathology of NASH caused by diet, including increased liver damage and fibrosis (38), while AMPK activation reduced NASH and HF by suppressing the production of fat, promoting the mitochondrial function in the liver, and inhibiting the proliferation of HSCs (39). AMPK is also considered the target of SCFAs involved in the alleviation of metabolic disease. Some studies reported that enterocytes exposed to acetate induced a marked increase in phosphorylated AMPKα and ameliorated lipid metabolism (30). These observations suggested that AMPK might play a role in HSC suppression by acetate. The antibody-based array kits and Western blot revealed that the phosphorylation of AMPKα was reduced after TGF-β1 treatment, indicating that the low phosphorylation level of AMPKα is closely related to HSC activation. We observed that acetate restored the decreased phosphorylation level of AMPKα induced by TGF-β1 and regulated the intracellular ratio of AMP and ATP, which might be critical to induce AMPKα phosphorylation. Reportedly, acetate was converted to acetyl CoA with the formation of AMP by the catalytic action of cytosolic acetyl CoA synthetase (AceCS1), and it might lead to increased AMP/ATP ratio in the cytosol. An increase in AMP/ATP ratio in the cytosol activated AMPK (40), which was considered as the primary mechanism related to activation of AMPK by acetate. Based on the results of the phosphokinase array and Western blot, we observed that c-Jun was involved in acetate-alleviated LX2 cell activation. Also, acetate reduced the phosphorylation of c-Jun and thus reduced the expression of the protein. Moreover, the reduced expression of c-Jun could inhibit its binding with Smad2, which in turn affects the TGF-β1-induced transcription of α-SMA (41). Fu et al. (42) demonstrated that acetate reduces the phosphorylation of c-Jun in adipocytes. Although c-Jun signaling was found to participate in the process of acetate-inhibited HSCs activation, future studies should explore the detailed mechanisms underlying the roles of c-Jun in HF, especially in HSC activation.

PPARγ is a nuclear receptor expressed in vascular smooth muscle cells and HSCs. It plays a role in the transcriptional control of cell growth, differentiation, and liver fibrosis. Cross-regulation by PPARγ of key fibrogenic factor TGF-β1 signaling have been identified as a main mechanisms by which PPARγ inhibits liver fibrosis (43, 44). PPARγ has been shown to disrupt TGF-β1-activated stress-activated protein kinase/c-Jun N-terminal kinase signaling, leading to decreased Smad2/3 activity and myofibroblast activation (34, 43). Similarly, Choi et al. showed that Capsaicin can ameliorate hepatic fibrosis by inhibiting the TGF-β1/Smad pathway via PPARγ activation (45). Our study indicated that NaA can activate PPARγ by increasing the phosphorylation of AMPK or reducing the phosphorylation of c-Jun. Considering the credible relation between PPARγ and TGF-β1/Smad2 pathway, we concluded that NaA inhibited TGF-β1/Smad2 pathway via PPARγ activation. PPARγ was highly expressed in quiescent HSCs of the normal liver but stimulated the inhibited HSC proliferation during hepatic fibrogenesis by reducing the expression of α-SMA protein and *Col1a1* mRNA (46). Intriguingly, PPARγ agonists/ligands inhibit both HSCs activation and HF (47). Therefore, PPARγ status is a pivotal marker for the success of antifibrotic therapy. AMPK is a known upstream agonist of PPARγ, and the activation of AMPK upregulates PPARγ expression (48). Some studies showed that the activation of AMPK/PPARγ pathway was related to the alleviation of NAFLD in mice (49). Na et al. (50) reported that acetate activated AMPK by increasing the AMP/ATP ratio, thereby increasing the PPARγ expression for blood pressure control. In this study, we found that NaA inhibited TGF-β1-induced increase in α-SMA expression and Smad2 phosphorylation, and silenced PPARγ disrupted the effects of NaA. These results at least partially confirmed the involvement of PPARγ in the inhibition of LX2 cells activated by NaA. We also observed that the phosphorylation level of AMPKα was related to HSC activation and thus, speculated that NaA inhibited HSC activation via AMPK/PPARγ pathway. The detailed interactions between AMPK and PPARγ and the role of acetate in this process need to be elucidated further.

## Conclusion

The TGF-β1-activated LX2 cells were alleviated by the effects of three dominant SCFAs, acetate, propionate, and butyrate. Acetate showed the best efficacy in suppressing fibrogenic markers and key signaling pathways in LX2 cells. We also found that NaA enters the cells and inhibits LX2 cell activation independent of GPR43, the canonical receptor of acetate. NaA also restores the phosphorylation levels of AMPK that are reduced in activated HSCs, while c-Jun and PPARγ are involved in the progress of inhibiting the activation of HSCs. Next, we suggested that the AMPK/PPARγ/Smad2 pathway is a novel mechanism associated with NaA-mediated anti-fibrosis against the activation of HSCs. This study provides evidence with respect to the correlation between gut microbial metabolites and the occurrence and development of HF and the connection of gut-liver axis and proposed sodium acetate as a putative candidate for the regulation of HF.

## Data Availability Statement

The datasets presented in this study can be found in online repositories. The names of the repository/repositories and accession number(s) can be found in the article/Supplementary Material.

## Author Contributions

XZ, SG, and LZ: conceptualization and supervision. WL: data curation and writing—original draft preparation. WL, MD, and JG: investigation, methodology, and validation. WL and LZ: writing—reviewing and editing. All authors contributed to the article and approved the submitted version.

## Funding

The authors declare that this study received funding from National Natural Science Foundation of China (32072196) and the 111 Project from the Education Ministry of China (No. B18053).

## Conflict of Interest

XZ was employed by Inner Mongolia Dairy Technology Research Institute Co., Ltd. The remaining authors declare that the research was conducted in the absence of any commercial or financial relationships that could be construed as a potential conflict of interest.

## Publisher's Note

All claims expressed in this article are solely those of the authors and do not necessarily represent those of their affiliated organizations, or those of the publisher, the editors and the reviewers. Any product that may be evaluated in this article, or claim that may be made by its manufacturer, is not guaranteed or endorsed by the publisher.

## Supplementary Material

Supplementary Figure 1 HDAC had a restrictive ability in the process of NaA inhibited TGF-β1 induced HSCs activation. LX2 cells were treated with or without 2.5 ng/ml TGF-β1 and 1 mM of NaA for 48 h. Real-time PCR was used to evaluate the mRNA expression of *HDAC*. LX2 cells were treated as detailed in the section Materials and Methods. For all bar graphs, data are the mean ± SD, ^#^*p* < 0.05, as compared with CON, and ^*^*p* < 0.05, as compared with MOD. The significant difference was assessed using the one-way ANOVA followed by LSD post-tests. Control group (CON), group model cell treated with a TGF-β1 (MOD), sodium acetate (NaA), arbitrary unit (AU).

Supplementary Table 1 Primers used in real-time qPCR.

Supplementary Table 2 Design of GPR43 siRNA targeting sequences.

Supplementary Table 3 Design of PPARγ siRNA targeting sequences.

Supplementary Table 4 Comparsion of level of protein phosphorylation among groups.

## References

[1] Friedman SL. Hepatic stellate cells: protean, multifunctional, and enigmatic cells of the liver. Physiol Rev. (2008) 88:125–72. 10.1152/physrev.00013.2007 18195085 PMC2888531

[2] Elpek GO. Cellular and molecular mechanisms in the pathogenesis of liver fibrosis: an update. World J Gastroenterol. (2014) 20:7260–76. 10.3748/wjg.v20.i23.7260 24966597 PMC4064072

[3] Han X Wu Y Yang Q Cao G. Peroxisome proliferator-activated receptors in the pathogenesis and therapies of liver fibrosis. Pharmacol Ther. (2021) 222:107791. 10.1016/j.pharmthera.2020.107791 33321113

[4] Tripathi A Debelius J Brenner DA Karin M Loomba R Schnabl B. The gut-liver axis and the intersection with the microbiome. Nat Rev Gastroenterol Hepatol. (2018) 15:397–411. 10.1038/s41575-018-0011-z 29748586 PMC6319369

[5] Josan S Billingsley K Orduna J Park JM Luong R Yu L. Assessing inflammatory liver injury in an acute CCl4 model using dynamic 3D metabolic imaging of hyperpolarized 1-C-13 pyruvate. NMR Biomed. (2015) 28:1671–77. 10.1002/nbm.3431 26474216 PMC4720258

[6] Puche JE Saiman Y Friedman SL. Hepatic stellate cells and liver fibrosis. Compr. (2013) 3:1473–92. 10.1002/cphy.c120035 24265236

[7] Rao HY Wei L Li J Zhang LF Chen HY Zhu LM. Liver fibrosis and hepatic stellate cells improvement of chronic hepatitis C patients by interferon-β-1α with or without sustained viral response. Hepato Gastroenterol. (2009) 56:328–34. 19579592

[8] Mazzotti A Caletti MT Sasdelli AS Brodosi L Marchesini G. Pathophysiology of non-alcoholic fatty liver disease: lifestyle-gut-gene interaction. Dig Dis. (2016) 34:3–10. 10.1159/000447275 27548720

[9] Poeta M Pierri L Vajro P. Gut-liver axis derangement in non-alcoholic fatty liver disease. Children. (2017) 4:66. 10.3390/children4080066 28767077 PMC5575588

[10] Le Roy T Llopis M Lepage P Bruneau A Rabot S Bevilacqua C. Intestinal microbiota determines development of non-alcoholic fatty liver disease in mice. Gut. (2013) 62:1787–94. 10.1136/gutjnl-2012-303816 23197411

[11] Cani PD. Human gut microbiome: hopes, threats and promises. Gut. (2018) 67:1716–25. 10.1136/gutjnl-2018-316723 29934437 PMC6109275

[12] Parekh PJ Balart LA Johnson DA. The influence of the gut microbiome on obesity, metabolic syndrome and gastrointestinal disease. Clin Transl Gastroenterol. (2015) 6:e91. 10.1038/ctg.2015.16 26087059 PMC4816244

[13] Boursier J Mueller O Barret M Machado M Fizanne L Araujo PF. The severity of non-alcoholic fatty liver disease is associated with gut dysbiosis and shift in the metabolic function of the gut microbiota. Hepatology. (2016) 63:764–75. 10.1002/hep.28356 26600078 PMC4975935

[14] Kakiyama G Pandak WM Gillevet PM Hylemon PB Heuman DM Daita K. Modulation of the fecal bile acid profile by gut microbiota in cirrhosis. J Hepatol. (2013) 58:949–55. 10.1016/j.jhep.2013.01.003 23333527 PMC3936319

[15] Gadallah SH Eissa S Ghanem HM Ahmed EK Hasanin AH Mahdy MM. Probiotic-prebiotic-synbiotic modulation of (*YAP1, LATS1* and *NF2* mRNAs*/miR-1205/lnc* RNA *SRD5A3-AS1*) panel in NASH animal model. Biomed Pharmacother. (2021) 140:111781. 10.1016/j.biopha.2021.111781 34090052

[16] Li M Zhou Y Zuo L Nie D Li X. Dietary fiber regulates intestinal flora and suppresses liver and systemic inflammation to alleviate liver fibrosis in mice. Nutrition. (2021) 81:110959. 10.1016/j.nut.2020.110959 33059126

[17] Besten G Bleeker A Gerding A Eunen K Havinga R Dijk TH. Short-chain fatty acids protect against high-fat diet-induced obesity via a PPAR-dependent switch from lipogenesis to fat oxidation. Diabetes. (2015) 64:2398–408. 10.2337/db14-1213 25695945

[18] Deng M Qu F Chen L Liu C Zhang M Ren F. SCFAs alleviated steatosis and inflammation in mice with NASH induced by MCD. J Endocrinol. (2020) 245:425–37. 10.1530/JOE-20-0018 32302970

[19] Takai A Kikuchi K Ichimura M Tsuneyama K Moritoki Y Matsumoto K. Fructo-oligosaccharides ameliorate steatohepatitis, visceral adiposity, and associated chronic inflammation via increased production of short-chain fatty acids in a mouse model of non-alcoholic steatohepatitis. BMC Gastroenterol. (2020) 20:46. 10.1186/s12876-020-01194-2 32103741 PMC7045471

[20] Takayama S Katada K Takagi T Iida T Ueda T Mizushima K. Partially hydrolyzed guar gum attenuates non-alcoholic fatty liver disease in mice through the gut-liver axis. World J Gastroenterol. (2021) 27:2160–76. 10.3748/wjg.v27.i18.2160 34025071 PMC8117741

[21] Jin Y Lu L Tu W Luo T Fu Z. Impacts of polystyrene microplastic on the gut barrier, microbiota and metabolism of mice. Sci Total Environ. (2019) 649:08–17. 10.1016/j.scitotenv.2018.08.353 30176444

[22] Wu JL Zou JY Hu ED Chen DZ Chen L Lu FB. Sodium butyrate ameliorates S100/FCA-induced autoimmune hepatitis through regulation of intestinal tight junction and toll-like receptor 4 signaling pathway. Immunol Lett. (2017) 190:169–76. 10.1016/j.imlet.2017.08.005 28811235

[23] Shi H Bi H Sun X Dong H Jiang Y Mu H. Antitumor effects of tubeimoside-1 in NCI-H1299 cells are mediated by microRNA-126-5p-induced inactivation of VEGF-A/VEGFR-2/ERK signaling pathway. Mol Med Rep. (2018) 17:4327–36. 10.3892/mmr.2018.8459 29363720 PMC5802206

[24] Luo X Li H Ma L Zhou J Guo X Woo SL. Expression of STING is increased in liver tissues from patients with NAFLD and promotes macrophage-mediated hepatic inflammation and fibrosis in mice. Gastroenterology. (2018) 155:1971. 10.1053/j.gastro.2018.09.010 30213555 PMC6279491

[25] Guo X Li H Xu H Halim V Thomas LN Woo SL. Disruption of inducible 6-phosphofructo-2-kinase impairs the suppressive effect of PPARγ activation on diet-induced intestine inflammatory response. J Nutr Biochem. (2013) 24:770–75. 10.1016/j.jnutbio.2012.04.007 22841546 PMC3584194

[26] Coady MJ Wallendorff B Bourgeois F Charron F Lapointe JY. Establishing a definitive stoichiometry for the Na^+^/monocarboxylate cotransporter SMCT1. Biophys J. (2007) 93:2325–31. 10.1529/biophysj.107.108555 17526579 PMC1965447

[27] Cai JW Zhang JT Tian Y Zhang LM Hatzakis E Krausz KW. Orthogonal comparison of GC MS and H-1 NMR spectroscopy for short chain fatty acid quantitation. Anal Chem. (2017) 89:7900–06. 10.1021/acs.analchem.7b00848 28650151 PMC6334302

[28] Huang W Man Y Gao C Zhou L Gu J Xu H. Short-chain fatty acids ameliorate diabetic nephropathy via GPR43-mediated inhibition of oxidative stress and NF-κB signaling. Oxid Med Cell Longevity. (2020) 2020:4074832. 10.1155/2020/4074832 32831998 PMC7422068

[29] Serrano HG Dolores CM Carlos MJ Corrales SV Carlos MJ Nunez LE. Phospho-kinase profile of colorectal tumors guides in the selection of multi-kinase inhibitors. Oncotarget. (2015) 6:31272–83. 10.18632/oncotarget.5211 26418718 PMC4741604

[30] Araujo JR Tazi A Burlen DO Vichier GS Nigro G Licandro H. Fermentation products of commensal bacteria alter enterocyte lipid metabolism. Cell Host Microbe. (2020) 27:358. 10.1016/j.chom.2020.01.028 32101704

[31] Tan YQ Chen HW Li J Wu QJ. Efficacy, chemical constituents, and pharmacological actions of radix paeoniae rubra and radix paeoniae alba. Front Pharmacol. (2020) 11:1054. 10.3389/fphar.2020.01054 32754038 PMC7365904

[32] Liu H Wang J He T Becker S Zhang G Li D. Butyrate: a double-edged sword for health? Adv Nutr. (2018) 9:21–29. 10.1093/advances/nmx009 29438462 PMC6333934

[33] Tolhurst G Heffron H Lam YS Parker HE Habib AM Diakogiannaki E. Short-chain fatty acids stimulate glucagon-like peptide-1 secretion via the G-protein-coupled receptor FFAR2. Diabetes. (2012) 61:364–71. 10.2337/db11-1019 22190648 PMC3266401

[34] Lee G You HJ Bajaj JS Joo SK Yu J Park S. Distinct signatures of gut microbiome and metabolites associated with significant fibrosis in non-obese NAFLD. Nat Commun. (2020) 11:4982. 10.1038/s41467-020-18754-5 33020474 PMC7536225

[35] Maslowski KM Vieira AT Ng A Kranich J Sierro F Yu D. Regulation of inflammatory responses by gut microbiota and chemoattractant receptor GPR43. Nature. (2009) 461:1282–U119. 10.1038/nature08530 19865172 PMC3256734

[36] Koh A De Vadder F Kovatcheva-Datchary P Backhed F. From dietary fiber to host physiology: short-chain fatty acids as key bacterial metabolites. Cell. (2016) 165:1332–45. 10.1016/j.cell.2016.05.041 27259147

[37] Kumar A Alrefai WA Borthakur A Dudeja PK. *Lactobacillus acidophilus* counteracts enteropathogenic *E. coli*-induced inhibition of butyrate uptake in intestinal epithelial cells. Am J Physiol Gastrointest Liver Physiol. (2015) 309:G602–G07. 10.1152/ajpgi.00186.2015 26272259 PMC4593819

[38] Zhao P Sun X Chaggan C Liao Z Wong KI He F. An AMPK-caspase-6 axis controls liver damage in non-alcoholic steatohepatitis. Science. (2020) 367:652. 10.1126/science.aay0542 32029622 PMC8012106

[39] Jiang S Li T Yang Z Yi W Di S Sun Y. AMPK orchestrates an elaborate cascade protecting tissue from fibrosis and aging. Ageing Res Revi. (2017) 38:18–27. 10.1016/j.arr.2017.07.001 28709692

[40] Yamashita H. Biological function of acetic acid-improvement in obesity and glucose tolerance by acetic acid in type 2 diabetic rats. Crit Rev Food Sci Nutr. (2016) 56:S171–S75. 10.1080/10408398.2015.1045966 26176799

[41] Liu F Shang YX. Sirtuin 6 attenuates epithelial-mesenchymal transition by suppressing the TGF-β1/Smad3 pathway and c-Jun in asthma models. Int Immunopharmacol. (2020) 82:106333. 10.1016/j.intimp.2020.106333 32143002

[42] Fu C Liu L Li F. Acetate alters the process of lipid metabolism in rabbits. Animal. (2018) 12:1895–902. 10.1017/S1751731117003275 29198236

[43] Zhao C Chen W Yang L Chen L Stimpson SA Diehl AM. PPARγ agonists prevent TGF β1/Smad3-signaling in human hepatic stellate cells. Biochem Biophys Res Commun. (2006) 350:385–91. 10.1016/j.bbrc.2006.09.069 17010940 PMC1760476

[44] Zhang F Lu Y Zheng S. Peroxisome proliferator-activated receptor- γ cross-regulation of signaling events implicated in liver fibrogenesis. Cell Signalling. (2012) 24:596–605. 10.1016/j.cellsig.2011.11.008 22108088

[45] Choi JH Jin SW Choi CY Kim HG Lee GH Kim YA. Capsaicin inhibits dimethylnitrosamine-induced hepatic fibrosis by inhibiting the TGF-β1/Smad pathway via peroxisome proliferator-activated receptor γ activation. J Agric Food Chem. (2017) 65:317–26. 10.1021/acs.jafc.6b04805 27991776

[46] Wang CY Liu Q Huang QX Liu JT He YH Lu JJ. Activation of PPARγ is required for hydroxysafflor yellow A of *Carthamus tinctorius* to attenuate hepatic fibrosis induced by oxidative stress. Phytomedicine. (2013) 20:592–99. 10.1016/j.phymed.2013.02.001 23523101

[47] Lee MF Liu ML Cheng AC Tsai ML Ho CT Liou WS. Pterostilbene inhibits dimethylnitrosamine-induced liver fibrosis in rats. Food Chem. (2013) 138:802–07. 10.1016/j.foodchem.2012.11.094 23411180

[48] Burns KA Vanden Heuvel JP. Modulation of PPAR activity via phosphorylation. Biochimica et Biophysica Acta. (2007) 1771:952–60. 10.1016/j.bbalip.2007.04.018 17560826 PMC2712836

[49] Liu E Wang X Li X Tian P Xu H Li Z. Co-exposure to multi-walled carbon nanotube and lead ions aggravates hepatotoxicity of non-alcoholic fatty liver via inhibiting AMPK/PPARγ pathway. Aging Us. (2020) 12:14189–204. 10.18632/aging.103430 32680977 PMC7425511

[50] Na L Chu X Jiang S Li C Li G He Y. Vinegar decreases blood pressure by down-regulating AT1R expression via the AMPK/PGC-1α/PPARγ pathway in spontaneously hypertensive rats. Eur J Nutr. (2016) 55:1245–53. 10.1007/s00394-015-0937-7 26476634

