# Supplementary figures and images for: Sodium Acetate Inhibit TGF-β1-Induced Activation of Hepatic Stellate Cells by Restoring AMPK or c-Jun Signaling
Source: Front Nutr. 2021 Sep 30;8:729583. doi: 10.3389/fnut.2021.729583 (PMC8515000; doi:10.3389/fnut.2021.729583)

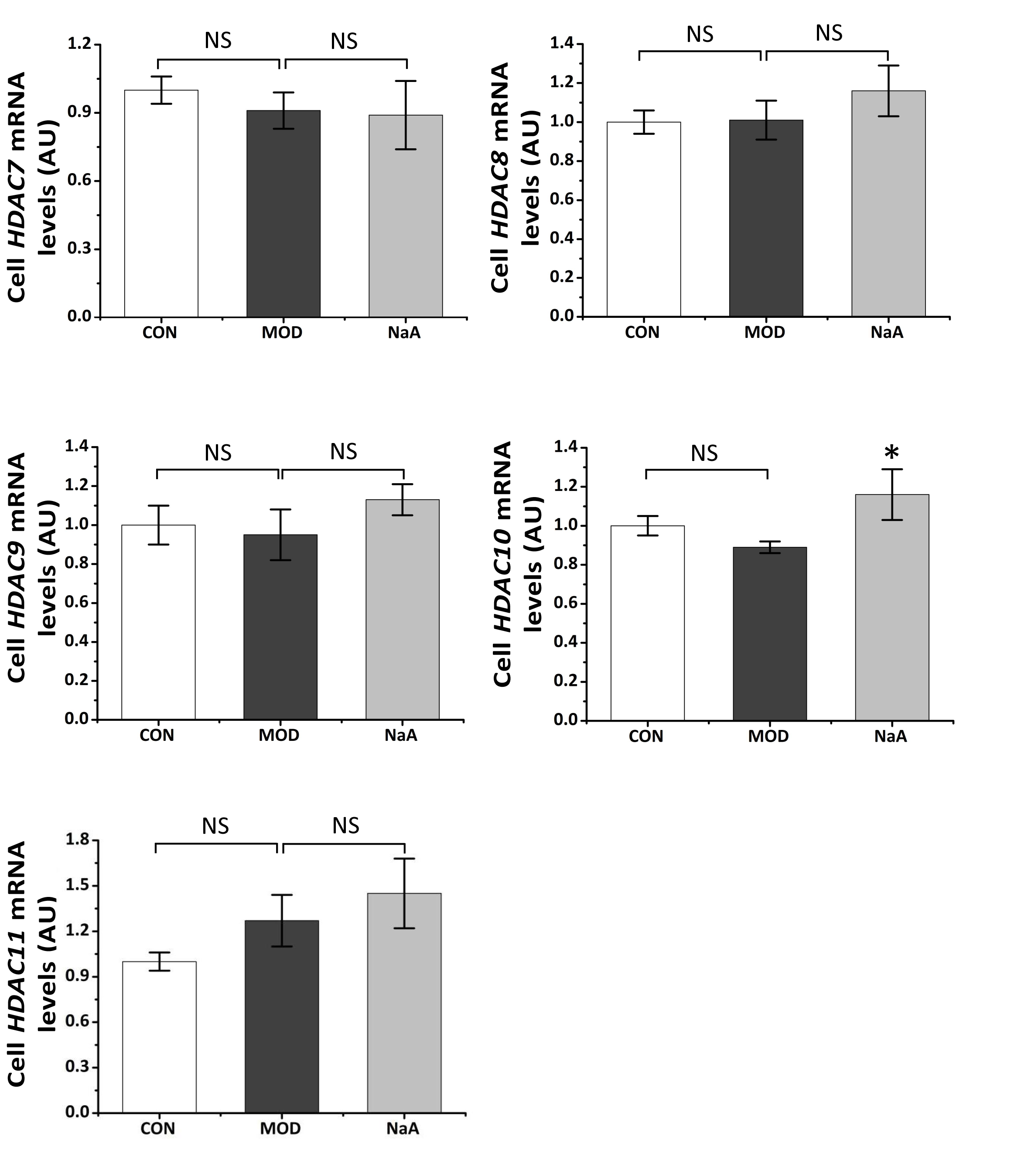

Supplement: Supplementary file 1 [file Image_2.TIF]

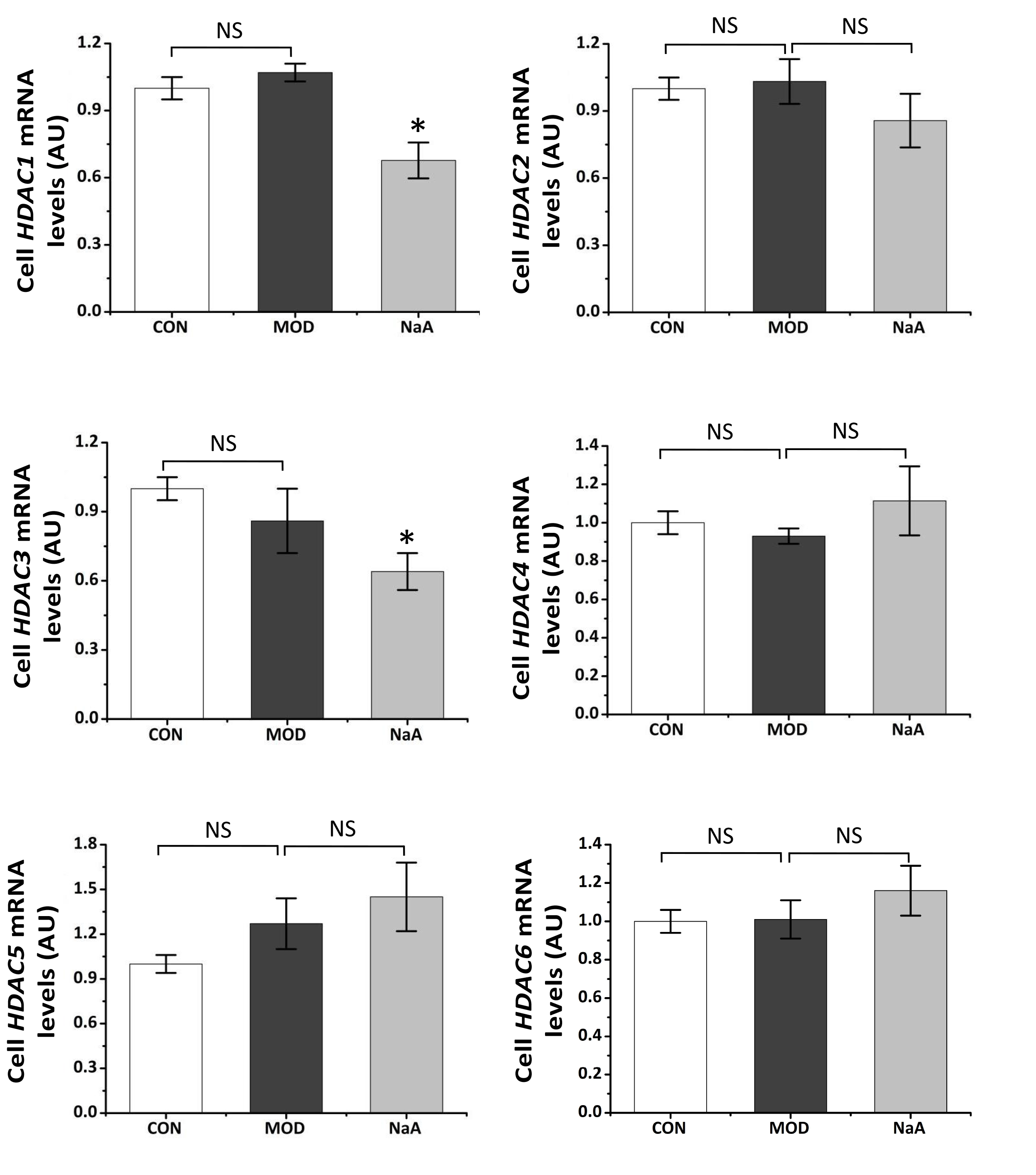

Supplement: Supplementary Figure 1 — HDAC had a restrictive ability in the process of NaA inhibited TGF-β1 induced HSCs activation. LX2 cells were treated with or without 2.5 ng/ml TGF-β1 and 1 mM of NaA for 48 h. Real-time PCR was used to evaluate the mRNA expression of HDAC. LX2 cells were treated as detailed in the section Materials and Methods. For all bar graphs, data are the mean ± SD, #p < 0.05, as compared with CON, and *p < 0.05, as compared with MOD. The significant difference was assessed using the one-way ANOVA followed by LSD post-tests. Control group (CON), group model cell treated with a TGF-β1 (MOD), sodium acetate (NaA), arbitrary unit (AU). [file Image_1.TIF]
