# Supplementary material for: Sodium Acetate Inhibit TGF-β1-Induced Activation of Hepatic Stellate Cells by Restoring AMPK or c-Jun Signaling
Source: Front Nutr. 2021 Sep 30;8:729583. doi: 10.3389/fnut.2021.729583 (PMC8515000; doi:10.3389/fnut.2021.729583)
Supplement: Supplementary Table 1 — Primers used in real-time qPCR. [file Table_1.DOC]

**Table S1**. Primers used in real-time qPCR.

| Gene (humen) | Number gene primer (5′ → 3′) |
| --- | --- |
| Colla1 | F：TGGTGACAAGGGTGAGACAG |
|  | R：CTCCAGAGGGACCTTGTTCA |
| Fn | F：CGGTGGCTGTCAGTCAAAG |
|  | R：AAACCTCGGCTTCCTCCATAA |
| Acta2 | F：ACCCACAATGTCCCCATCTA |
|  | R：AATAGCCACGCTCAGTCAGG |
| GPR43 | F：GCCTGGTGCTCTTCTTCATC |
|  | R：AGGTGGGACACGTTGTAAGG |
| PPARγ | F：GGGATCAGCTCCGTGGATCT |
|  | R：TGCACTTTGGTACTCTTGAAGTT |
| HDAC1 | F：CGCCCTCACAAAGCCAATG |
|  | R：CTGCTTGCTGTACTCCGACA |
| HDAC2 | F：ATGGCGTACAGTCAAGGAGG |
|  | R：TGCGGATTCTATGAGGCTTCA |
| HDAC3 | F：GCAAGGCTTCACCAAGAGTCT |
|  | R：AGATGCGCCTGTGTAACGC |
| HDAC4 | F：AGCGTCCGTTGGATGTCAC |
|  | R：CCTTCTCGTGCCACAAGTCT |
| HDAC5 | F：GGTGTGGTCTACGACACGTTC |
|  | R：GATCCGCTCGCACTTGCTAA |
| HDAC6 | F：AAGAAGACCTAATCGTGGGACT |
|  | R：GCTGTGAACCAACATCAGCTC |
| HDAC7 | F：GGCGGCCCTAGAAAGAACAG |
|  | R：CTTGGGCTTATAGCGCAGCTT |
| HDAC8 | F：TCGCTGGTCCCGGTTTATATC |
|  | R：TACTGGCCCGTTTGGGGAT |
| HDAC9 | F：ATGGTTTCACAGCAACGCATT |
|  | R：ACCTTGCCTAAGCGTCTGC |
| HDAC10 | F：AGTGCCCTAGAGTCCATCCAG |
|  | R：CACAGCGGTCACATCTTGCT |
| HDAC11 | F：ACCCAGACAGGAGGAACCATA |
|  | R：TGATGTCCGCATAGGCACAG |
| GAPDH | F：CTGGGCTACACTGAGCACC |
|  | R：AAGTGGTCGTTGAGGGCAATG |

F:forward primer; R:reverse primer.

**Table S2.** Design of GPR43 siRNA targeting sequences

| Gene (humen) | Number gene primer (5′ → 3′) |
| --- | --- |
| siGPR43-1 | F：GCAUCAGCAUCGAGCGCUATT |
|  | R：UAGCGCUCGAUGCUGAUGCTT |
| siGPR43-2 | F：GAAAUUACCUGCUACGAGATT |
|  | R：UCUCGUAGCAGGUAAUUUCTT |
| siGPR43-3 | F：ACGCAGAGGCAAAGACACATT |
|  | R：UGUGUCUUUGCCUCUGCGUTT |

F:forward primer; R:reverse primer.

**Table S3**. Design of PPARγ siRNA targeting sequences

| Gene (humen) | Number gene primer (5′ → 3′) |
| --- | --- |
| siPPARγ-1 | F：UCCGUGGAUCUCUCCGUAAUGTT |
|  | R：CAUUACGGAGAGAUCCACGGATT |
| siPPARγ-2 | F：GAGAUCACAGAGUAUGCCAAATT |
|  | R：UUUGGCAUACUCUGUGAUCUCTT |
| siPPARγ-3 | F：GACAGCGACUUGGCAAUAUUUTT |
|  | R：AAAUAUUGCCAAGUCGCUGUCTT |

F:forward primer; R:reverse primer.

| **Table S4**. Comparsion of level of protein phosphorylation among groups. | | | | | | | | | | |
| --- | --- | --- | --- | --- | --- | --- | --- | --- | --- | --- |
| protein |  | |  | | CON vs MOD | |  | | MOD vs NaA | |
|  | CON^a^ | | MOD^b^ | | Significant up-regulated or down-regulated by TGF-β1^d^ | | NaA^c^ | | Significant up-regulated or down-regulated by NaA^e^ | |
| p38alpha | | 0.130678±0.0382512 | | 0.078418±0.0076669 | |  | | 0.086336±0.0162121 | |  |
| ERK1/2 | | 0.120857±0.0066147 | | 0.110873±0.0009604 | | ↓ | | 0.105129±0.0017595 | | ↓ |
| JNK 1/2/3 | | 0.149869±0.0079273 | | 0.163301±0.0034264 | | ↑ | | 0.180225±0.0023297 | | ↑ |
| GSK-3alpha/beta | | 0.189317±0.005394 | | 0.195358±0.0071738 | |  | | 0.222953±0.0088907 | | ↑ |
| EGF R | | 0.07744±0.0070947 | | 0.070588±0.0087366 | |  | | 0.085301±0.0108686 | |  |
| MSK1/2 | | 0.096338±0.0001121 | | 0.081422±0.0024217 | |  | | 0.09739±0.0072612 | | ↑ |
| AMPKalpha1 | | 0.081337±0.0017538 | | 0.075436±0.0014292 | | ↓ | | 0.084014±0.0003917 | | ↑ |
| Akt 1/2/3 | | 0.078233±0.0069118 | | 0.079275±0.0039166 | |  | | 0.086807±0.0032219 | |  |
| TOR | | 0.055195±0.0016351 | | 0.059965±0.000139 | | ↑ | | 0.06461±0.0012569 | | ↑ |
| CREB | | 0.112944±0.004991 | | 0.124056±0.0024695 | | ↑ | | 0.13869±0.0020315 | | ↑ |
| HSP27 | | 0.051621±0.0035778 | | 0.060049±0.0003913 | | ↑ | | 0.062241±0.0022911 | |  |
| AMPKalpha2 | | 0.06302±0.0029367 | | 0.07484±0.0021158 | | ↑ | | 0.08457±0.0008421 | | ↑ |
| beta-Catenin | | 0.042408±0.005014 | | 0.04734±0.0026165 | |  | | 0.053834±0.0023037 | |  |
| Src | | 0.077228±0.0022789 | | 0.080032±0.0059071 | |  | | 0.09657±0.0033858 | | ↑ |
| Lyn | | 0.038486±0.0003906 | | 0.043231±0.0005424 | | ↑ | | 0.04426±0.0016552 | |  |
| Lck | | 0.028785±0.0013515 | | 0.033543±0.0020921 | |  | | 0.033457±0.0017985 | |  |
| STAT2 | | 0.081058±0.0008618 | | 0.100554±0.0000081 | | ↑ | | 0.110999±0.0039798 | | ↑ |
| STAT5a | | 0.035997±0.0043758 | | 0.044787±0.0044575 | |  | | 0.046963±0.0040511 | |  |
| Fyn | | 0.037925±0.0019679 | | 0.042384±0.0006191 | | ↑ | | 0.049494±0.0017655 | | ↑ |
| Yes | | 0.079228±0.0012875 | | 0.080909±0.0003941 | |  | | 0.08504±0.0035136 | |  |
| Fgr | | 0.025537±0.0027914 | | 0.027804±0.0026347 | |  | | 0.027448±0.00203 | |  |
| STAT6 | | 0.063199±0.0013667 | | 0.073294±0.0012603 | | ↑ | | 0.077538±0.0026182 | |  |
| STAT5b | | 0.032229±0.0018215 | | 0.036948±0.0025838 | |  | | 0.039082±0.0042639 | |  |
| Hck | | 0.066963±0.0105808 | | 0.085064±0.0057378 | |  | | 0.084339±0.0058461 | |  |
| Chk-2 | | 0.090043±0.000448 | | 0.0978±0.0002921 | |  | | 0.103027±0.005939 | |  |
| FAK | | 0.121024±0.0079798 | | 0.127679±0.0036215 | |  | | 0.124235±0.0016427 | |  |
| PDGF Rbeta | | 0.039256±0.0005951 | | 0.043763±0.0003048 | | ↑ | | 0.045596±0.0002102 | | ↑ |
| STAT5a/b | | 0.045337±0.0005829 | | 0.053824±0.0021232 | | ↑ | | 0.055841±0.0034064 | |  |
| PRAS40 | | 0.089936±0.0113479 | | 0.095954±0.0172256 | |  | | 0.099513±0.0208422 | |  |
| p53 | | 0.051005±0.000483 | | 0.050802±0.0020625 | |  | | 0.054523±0.0006803 | | ↑ |
| Akt123 | | 0.074174±0.0006337 | | 0.075092±0.0013937 | |  | | 0.070794±0.0022713 | |  |
| p53a | | 0.063056±0.0008553 | | 0.05994±0.0012645 | |  | | 0.059946±0.0002307 | |  |
| p70 S6 Kinase | | 0.050461±0.0011657 | | 0.052731±0.0026236 | |  | | 0.044249±0.002676 | | ↓ |
| p53b | | 0.03056±0.0015136 | | 0.026179±0.0000358 | |  | | 0.021466±0.0004898 | | ↓ |
| c-Jun | | 0.086637±0.0006926 | | 0.094994±0.0027669 | | ↑ | | 0.081272±0.0018497 | | ↓ |
| RSK1/2/3 | | 0.03871±0.0019653 | | 0.042263±0.0028627 | |  | | 0.031356±0.0011217 | | ↓ |
| eNOS | | 0.023422±0.0001012 | | 0.020401±0.0003845 | | ↓ | | 0.018451±0.0003713 | | ↓ |
| STAT3 | | 0.12127±0.0015267 | | 0.125168±0.0038964 | |  | | 0.116857±0.001251 | | ↓ |
| p27 | | 0.040025±0.0043555 | | 0.041607±0.004062 | |  | | 0.032593±0.0029326 | |  |
| PLC-gamma1 | | 0.056284±0.0007866 | | 0.052876±0.0005855 | | ↓ | | 0.041465±0.000506 | | ↓ |
| STAT3 | | 0.177147±0.0035106 | | 0.173794±0.0019204 | | ↓ | | 0.144869±0.0014433 | |  |
| WNK1 | | 0.144294±0.0032003 | | 0.157237±0.0044921 | |  | | 0.127063±0.0066804 | | ↓ |
| PYK2 | | 0.073791±0.001403 | | 0.070692±0.0000201 | | ↓ | | 0.059523±0.0005073 | | ↓ |
| HSP60 | | 0.16798±0.0047046 | | 0.172174±0.0060322 | |  | | 0.156769±0.0001679 | | ↓ |

^a^CON, control group.

^b^MOD, group model cell treated with TGF-β1.

^c^NaA, group model cell treated with TGF-β1 and NaA.

^d^The level of protein phosphorylation changed significantly between CON and MOD were marked (*VIP* > 1, *p* < 0.1). ↑ indicated the level of protein. phosphorylation was up-regulated after TGF-β1 intervention. ↓ indicated the level of protein phosphorylation was down-regulated after TGF-β1 intervention.

^e^The level of protein phosphorylation changed significantly between MOD and NaA were marked (*VIP* > 1, *p* < 0.1). ↑ indicated the level of protein. phosphorylation was up-regulated after NaA intervention. ↓ indicated the level of protein phosphorylation was down-regulated after NaA intervention.
